# RIG: Recalibration and Interrelation of Genomic Sequence Data with the GATK

**DOI:** 10.1534/g3.115.017012

**Published:** 2015-02-13

**Authors:** Ryan F. McCormick, Sandra K. Truong, John E. Mullet

**Affiliations:** * Interdisciplinary Program in Genetics, Texas A&M University, College Station, Texas 77843; † Biochemistry & Biophysics Department, Texas A&M University, College Station, Texas 77843

**Keywords:** GATK, variant calling, genomic sequence data

## Abstract

Recent advances in variant calling made available in the Genome Analysis Toolkit (GATK) enable the use of validated single-nucleotide polymorphisms and indels to improve variant calling. However, large collections of variants for this purpose often are unavailable to research communities. We introduce a workflow to generate reliable collections of single-nucleotide polymorphisms and indels by leveraging available genomic resources to inform variant calling using the GATK. The workflow is demonstrated for the crop plant *Sorghum bicolor* by (i) generating an initial set of variants using reduced representation sequence data from an experimental cross and association panels, (ii) using the initial variants to inform variant calling from whole-genome sequence data of resequenced individuals, and (iii) using variants identified from whole-genome sequence data for recalibration of the reduced representation sequence data. The reliability of variants called with the workflow is verified by comparison with genetically mappable variants from an independent sorghum experimental cross. Comparison with a recent sorghum resequencing study shows that the workflow identifies an additional 1.62 million high-confidence variants from the same sequence data. Finally, the workflow’s performance is validated using *Arabidopsis* sequence data, yielding variant call sets with 95% sensitivity and 99% positive predictive value. The Recalibration and Interrelation of genomic sequence data with the GATK (RIG) workflow enables the GATK to accurately identify genetic variation in organisms lacking validated variant resources.

The decreasing cost of high-throughput sequencing has led to a proliferation of template preparation methods and sequence data (Sims *et al.* 2014). The abundance of sequence data has motivated an interest in leveraging available data to identify genetic variation, and software development has kept pace with this demand as exemplified by the Broad Institute’s open-source Genome Analysis Toolkit (GATK). The GATK can integrate evidence for variants from multiple samples with joint genotyping, and it enables the use of validated single-nucleotide polymorphisms (SNPs) and indels to improve the accuracy of variant calling. Additionally, the GATK’s methods are implemented in a manner amenable to reads originating from a variety of template preparation methods and sequencing platforms (Depristo *et al.* 2011). However, many research communities lack the large, validated collections of SNPs and indels necessary for the GATK’s Best Practices procedures because of the investment necessary to produce and curate such collections (Van Der Auwera *et al.* 2013). As an alternative to large-scale variant validation studies, we developed the Recalibration and Interrelation of genomic sequence data with the GATK (RIG) workflow to integrate information from multiple genomic sources and identify reliable sets of variants.

The GATK has gained extensive adoption in the human genomics research community due in part to the methods it uses to account for known error sources during variant calling; accounting for these error sources enables the GATK to consistently outperform other modern variant callers in benchmarking studies (Depristo *et al.* 2011; Nekrutenko and Taylor 2012; Liu *et al.* 2013; Pirooznia *et al.* 2014). Multiple sources of error exist, including incomplete or incorrect reference assemblies, erroneous realignment of reads to the reference genome (particularly in low complexity regions and around indels), inaccurate base quality scores, and suboptimal variant filtration parameters (Depristo *et al.* 2011; Li 2014). Features of the GATK address a number of these error sources, and we briefly describe three of the features most relevant to the design of the RIG workflow. The first feature is Base Quality Score Recalibration (BQSR), where the base quality scores assigned by the sequencer are corrected with scores empirically determined from the read group data using validated variants; these recalibrated scores more accurately reflect the true reliability of the base calls, thus correcting biases introduced by sequencing platforms (Li *et al.* 2004). The second feature is the GATK’s joint genotyping methodology that can integrate the evidence for a variant from many samples on reasonable time scales; this allows data from thousands of samples to be considered when evaluating the existence of a variant. The third feature is Variant Quality Score Recalibration (VQSR), where raw variant calls are assigned probabilities of being true variants based on the behavior of training variants in the raw variant calls using machine learning techniques (McKenna *et al.* 2010; Depristo *et al.* 2011; Van Der Auwera *et al.* 2013). These probabilities allow users to decide which variants to use in downstream analyses based on desired levels of specificity and sensitivity, where high specificity indicates a low false-positive rate, and high sensitivity indicates a low false-negative rate. Two of these three features, BQSR and VQSR, require a collection of reliable variants to function effectively, and their benefits are inaccessible without such a resource.

Although many research communities lack large, validated resources of known SNPs and indels, some communities, namely agricultural research communities, often have access to a variety of genomic data sources that can be used to identify reliable genetic variants for use with the RIG workflow. Two characteristics influence the optimal use of these data sources with the RIG workflow: (i) the method used to produce the source’s raw data from which variants are called, and (ii) the experimental design behind the source. First, many methods are available to produce the raw data from which variants are called, including reduced representation sequencing, whole-genome sequencing (WGS), SNP chips, Sanger sequencing, and RNA sequencing. Variants identified from two different methods can be considered more reliable than those identified in only one, as they are less likely to be artifacts introduced by a specific method. The RIG workflow can take advantage of multiple data sources by using variants found from one data source to inform the analysis of a second, read-based data source; by providing variants obtained from orthogonal methods, the reliability of variant resources used in BQSR and VQSR can be improved. Second, the experimental design behind the source also influences the reliability of the variants obtained from the source. Two experimental design elements influencing the reliability of a genomic variant are (i) if the variant segregates according to Mendelian expectations, and (ii) how often the variant is observed in independent samples. Genotyping large experimental crosses provides variants where Mendelian violations can be identified and the variants are observed at high frequencies in independent samples; as such, experimental crosses represent one of the most reliable sources of genetic variants. Association panels or population samples can also provide a reliable source of variants given a sufficiently large sample size and minor allele frequency. When Mendelian violations cannot be identified or when sample sizes are small (as is common with resequencing designs), variants are considered less reliable. For our use case with sorghum, we (i) generated an initial set of variants using reduced representation sequence data from an experimental cross and association panels, (ii) used the initial variants to inform variant calling from WGS data of resequenced individuals, and (iii) used variants identified from WGS data for recalibration of the reduced representation sequence data. By considering the method used to produce the raw data from which variants are called and the experimental design behind the data source, available genomic sequence data can be optimally leveraged to improve variant calling and subsequent analyses.

Here we present the RIG workflow to formalize the process of incorporating available genomic sequence resources when calling SNPs and indels with the GATK. The RIG workflow is designed to leverage available genomic data in a manner that maximizes the information available to joint genotyping and to produce collections of reliable variants sufficiently large to perform BQSR and VQSR; this provides the benefits of the GATK’s methods even in the absence of a large collection of validated variants, and it is readily applicable to organisms with a reference genome sequence and moderate sequence data resources. As an example, we describe the RIG workflow using *Sorghum bicolor* sequence data and show that it readily interrelates reduced representation and WGS data to generate variant calls. We evaluate the performance of the RIG workflow for sorghum sequence data using a collection of genetically validated variants, and we compare the output of the RIG workflow with variant calls from a recent sorghum study. Finally, we validate the workflow with *Arabidopsis* sequence data and show that high sensitivity and specificity is readily achieved.

## Materials and Methods

### Sorghum analyses

**Figure 1:**
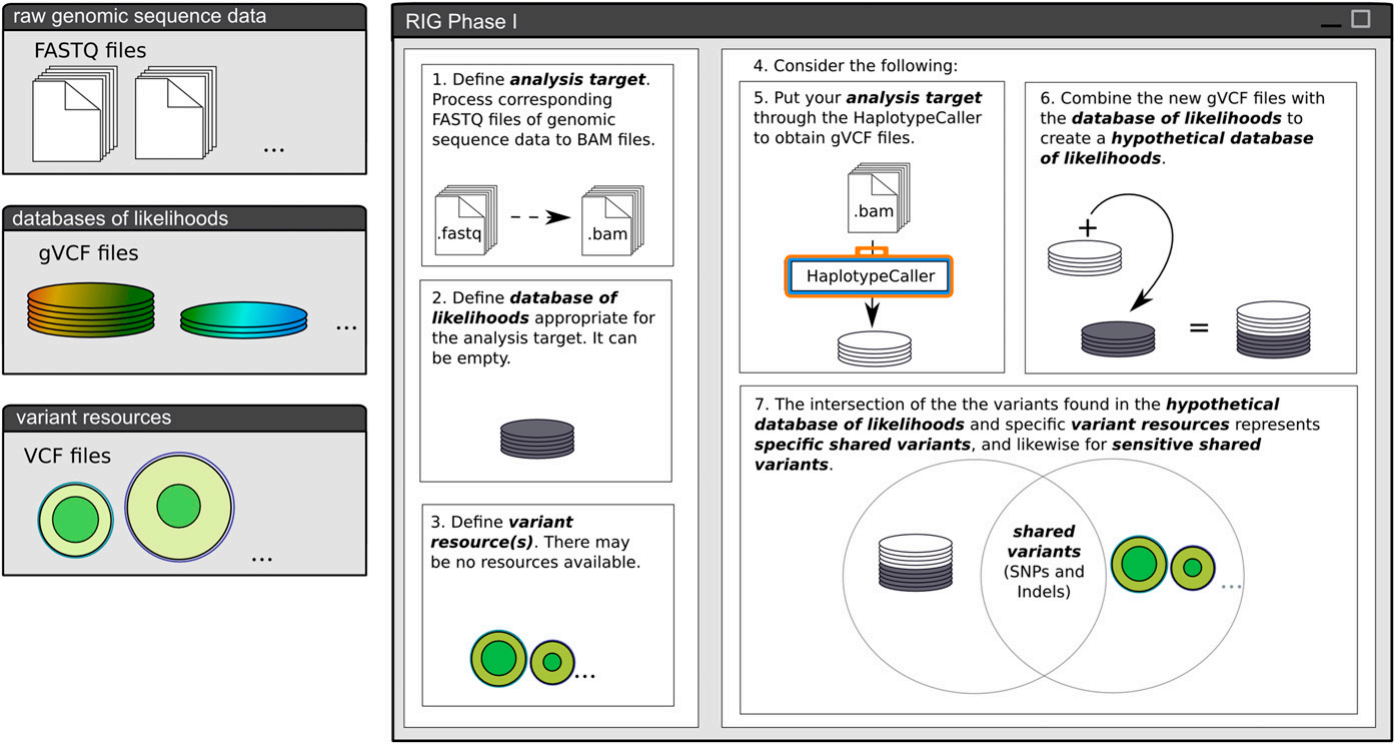
Phase I of the RIG workflow. Phase I of the RIG workflow defines the five entities necessary for the execution of Phase II. Once the first three entities, the analysis target, database of likelihoods, and variant resource(s) are defined, the user considers a hypothetical case based on those first three entities to estimate the contents of the remaining two: the hypothetical database of likelihoods and the shared variants. If a user is unable to make a prediction regarding the latter two entities, the entities can either be treated as empty sets, or the user can use the GATK to carry out the necessary procedures to generate an estimate. Once all five entities are defined, the user can proceed to Phase II. RIG, Recalibration and Interrelation of genomic sequence data with the GATK; GATK, Genome Analysis Toolkit.

**Figure 2:**
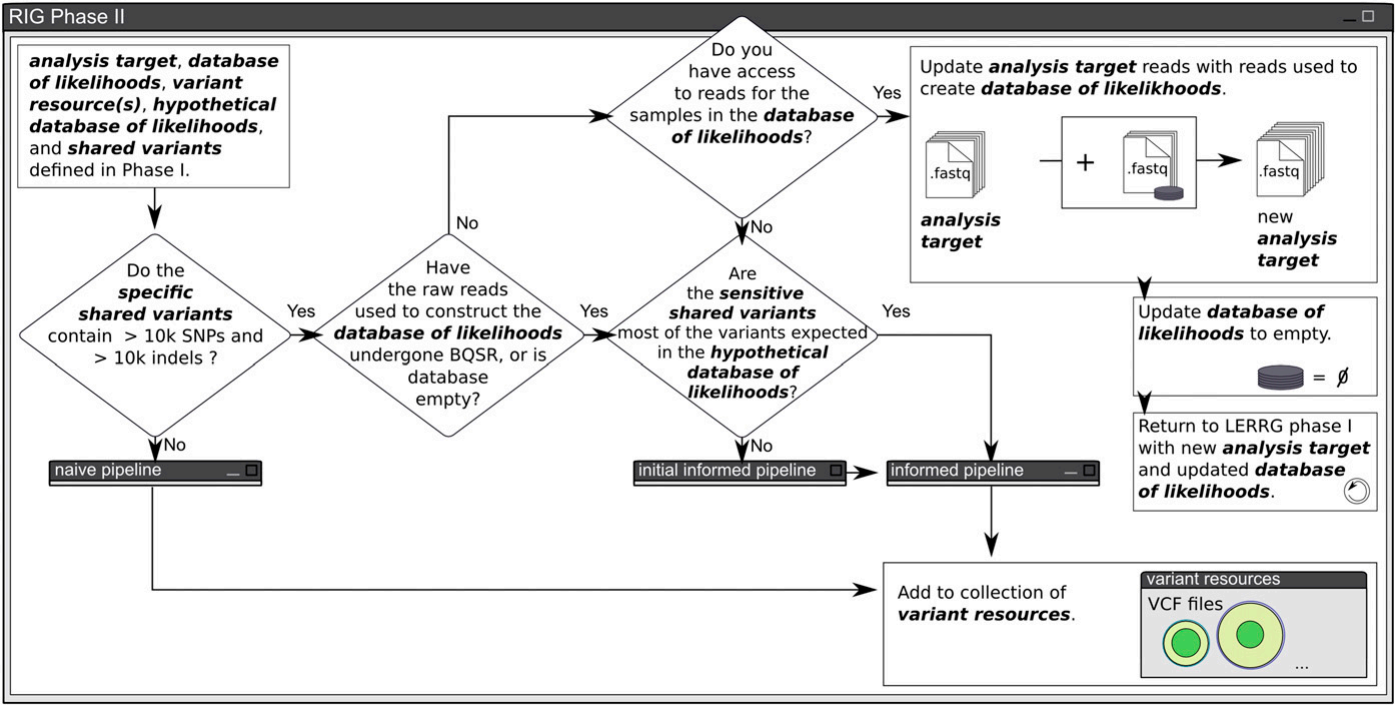
Phase II of the RIG workflow. Phase II of the RIG workflow determines whether VQSR, BQSR, or both are appropriate, given the entities defined in Phase I. The workflow always proceeds through an analysis pipeline, characterized as the naive, the initial informed, and the informed pipelines shown in Figure 3. The end result of the workflow is the production of a variant resource that can be used in future analyses. RIG, Recalibration and Interrelation of genomic sequence data with the GATK; VQSR, Variant Quality Score Recalibration; BQSR, Base Quality Score Recalibration.

**Figure 3:**
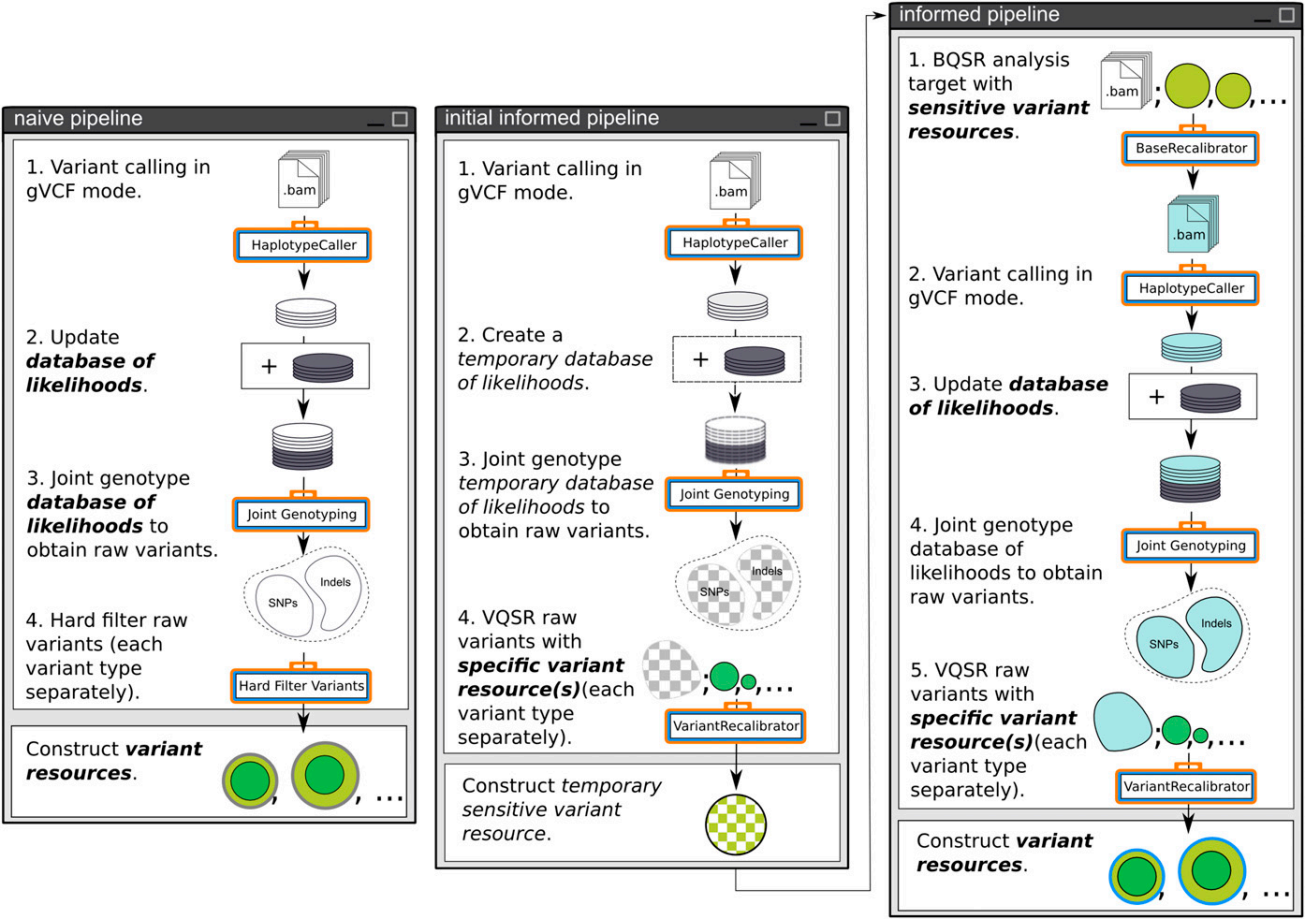
RIG pipelines. These are analysis pipelines that are traversed as part of Phase II of the RIG workflow. They correspond to cases where neither BQSR nor VQSR are appropriate (naive pipeline), where only VQSR is appropriate (initial informed pipeline), or where both BQSR and VQSR are appropriate (informed pipeline). When traversed, the informed pipeline emulates the GATK’s Best Practices (Van Der Auwera *et al.* 2013). RIG, Recalibration and Interrelation of genomic sequence data with the GATK; BQSR, Base Quality Score Recalibration; VQSR, Variant Quality Score Recalibration; GATK, Genome Analysis Toolkit.

**Figure 4:**
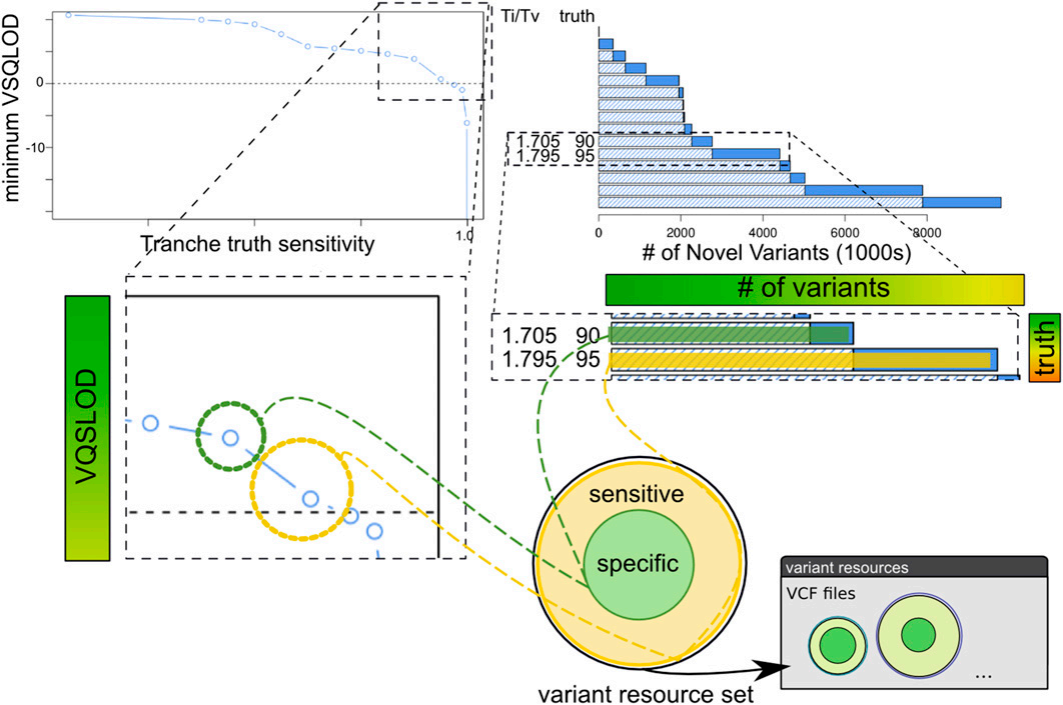
Construction of variant resources. After VQSR, multiple tranches are evaluated to choose specific and sensitive sets of variants for use in downstream analyses and to designate as variant resources. Tranches correspond to VQSLOD cutoffs above which a specified percentage of the variants designated as truth during VQSR are retained in the tranche. For example, a 95% tranche indicates the VQSLOD cutoff at which 95% of the variants designated as truth during VQSR would be retained. Accordingly, lower tranche percentages have greater specificity, lesser sensitivity, and contain fewer variants, and lower percentage tranches are subsets of greater percentage tranches. Here we show a 90% tranche being chosen as the specific variant resource and the 95% tranche being chose as the sensitive variant resource; both are subsequently added to the collection of variant resources. Note that the specific variant resource generated here is a subset of the sensitive variant resource. VQSR, Variant Quality Score Recalibration; VQSLOD, logarithm of odds ratio that a variant is real *vs.* not under the trained Gaussian mixture model;

The RIG workflow described in the *Results* section was designed as a generalization of our use cases in leveraging existing *Sorghum bicolor* genomic resources to take advantage of the GATK’s strengths. Here we describe the process of transitioning from exclusive use of the naive pipeline to use of the initial informed and informed pipelines as an example of executing the RIG workflow and constructing variant resources (Figure 1, Figure 2, Figure 3, and Figure 4).

At the time of transitioning from the naive pipeline to the initial informed and informed pipelines with *Sorghum bicolor* sequence data, we had access to reduced representation sequence data generated internally by Digital Genotyping using the restriction enzyme NgoMIV and a collection of WGS data generated from multiple groups (Zheng *et al.* 2011; Evans *et al.* 2013; Mace *et al.* 2013; Morishige *et al.* 2013). Using reduced representation sequence data for a 423 member recombinant inbred line population, we used the naive pipeline to produce variant calls (Burow *et al.* 2011; Truong *et al.* 2014). Preprocessing of the reads prior to variant calling, including read-mapping to version 1 of the *Sorghum bicolor* reference assembly, was performed using BWA and Picard (Paterson *et al.* 2009; Li and Durbin 2010; Picard Team 2014). Variants were genetically mapped with R/qtl, and variants segregating as expected in these calls were used to create a Family Reference Variant Resource that contained 6849 SNPs and 2164 indels (Broman *et al.* 2003). The Family Reference Variant Resource was considered a highly specific variant resource. Of note, the genetic positions of markers found in this manner are also being used to anchor unplaced super contigs in the *Sorghum bicolor* reference genome assembly (J. Schmutz, personal communication). Similarly, reduced representation sequence data for 733 sorghum germplasm samples processed with the naive pipeline were used to produce a Population Reference Variant Resource containing 62,022 SNPs and 20,801 indels. This variant resource was considered specific because we enforced a genotyping rate of 60% and a minor allele frequency of 0.05. The hard filtering parameters that we use in the naive pipeline for reduced representation sequence data can be found within the implementation on GitHub at https://github.com/MulletLab/RIG.

We sought to use these variants found in reduced representation sequence data to improve the analysis of WGS data of the 49 publicly available WGS data sources (Zheng *et al.* 2011; Evans *et al.* 2013; Mace *et al.* 2013). To do this, we chose 10 individuals from the 49 that represented diverse sorghum germplasm accessions. We attempted to maximize diversity so that the sensitive variant resource constructed after the initial informed and informed pipelines had been executed would include many of the variants present in the next group of individuals for BQSR; this enabled use of the informed pipeline in the following iterations as the remaining 39 samples were processed (individuals were processed 10 at a time due to hard disk space limitations).

With the Family and Population Reference Variant Resources and the 10 WGS samples as analysis targets, we met the requirements for VQSR but not BQSR (Figure 1, Figure 2, and Figure 3). As such, we followed the initial informed pipeline. For VQSR, the Family and Population Reference Variant Resources were both designated as truth, training, and known variants, and priors set to 15.0 and 7.0, respectively. Although these settings worked for our use case, they may not always be applicable; however, we typically follow these general rules: only highly specific variant resources should be designated as truth; variant resources designated as training do not need to be highly specific, but their priors should be set accordingly; all resources designated as truth should also be designated as training; and resources designated as truth and training can also be designated as known. Other details along with the annotations used for training the SNP and indel Gaussian mixture models can be found with the implementation on GitHub at https://github.com/MulletLab/RIG.

Generating variant resources following VQSR is a highly user-driven process that depends largely on the user’s confidence in the variant resources designated as truth for VQSR, and it requires examining multiple tranches resulting from VQSR (Figure 4). Tranches represent cutoffs based on variant resources designated as truth during VQSR, and they are generated by considering the VQSLOD scores (logarithm of odds ratio that a variant is real *vs.* not under the trained Gaussian mixture model) of truth variants that are present in the recalibrated raw variants. For example, if 90% of the truth variants found in the raw variants had a VQSLOD score over 1.5, then the 90% tranche would contain all variants in the raw variants that had a VQSLOD score over 1.5. We typically pick two tranches after VQSR, a specific tranche and a sensitive tranche, by examining the behavior of VQSLOD scores of multiple tranches (Figure 4). Specific tranches typically come from tranches where the VQSLOD score changes by small amounts even as the tranche percentage is decreased, and sensitive tranches are typically a non-negative VQSLOD score tranche that is more inclusive than the specific tranche.

Having generated a temporary sensitive variant resource from the initial 10 WGS samples using the initial informed pipeline, we proceeded down the informed pipeline with those 10 samples to generate a sensitive Whole-Genome Sequence Variant Resource. We then iteratively processed the remaining 39 samples in groups of 10 (9 on the final iteration) using the informed pipeline and updating the Whole-Genome Sequence Variant Resource each iteration; we continued to use only the Family and Population Reference Variant Resources for VQSR (to enforce that variants designated as truth for VQSR had been identified using a different sequencing template preparation method), and we used the newest sensitive Whole-Genome Sequence Variant Resource for BQSR. Upon completion of all 49 genomes, we used the newest Whole-Genome Sequence Variant Resources for BQSR and VQSR of the association panel data (sensitive for BQSR and specific for VQSR) to generate the Sensitive Population Reference Variant Resource (97.5% tranche) that was used for the genome-wide association study.

The IF set used to examine the recalibration of WGS variants was constructed from a second biparental recombinant inbred line population (Xu *et al.* 2000). Variants from this population were generated in the same fashion as the Family Reference Variant Resource (*i.e.*, using NgoMIV Digital Genotyping, the naive pipeline, and checking for Mendelian segregation in R/qtl). The Raw, Sensitive, and Specific sets used in the comparison with the IF set were derived from the 100% tranche, the 95% tranche, and 75% tranche of the recalibrated WGS variants (Supporting Information, Table S1 and Table S2, and Figure S1). The Raw, Sensitive, and Specific sets used for comparison with the Gramene42-Mace2013 set originate in the same manner, but excluded indels, SNPs on super contigs, and variants not found in 1 of the 47 samples to be comparable with the Gramene42-Mace2013 set. Variants and genotypes for 171 individuals from the Sensitive Population Reference Variant Resource were used with downstream analysis tools to perform the association mapping described and depicted in Figure S2 and Table S3.

### *Arabidopsis* analyses

Publicly available WGS for five accessions (ICE50 ICE134, ICE150, ICE213, and Leo-1) from Cao *et al.* (2011) were processed using the naive pipeline and stringently hard filtered (parameters available on GitHub). Publicly available Sanger sequence for 20 accessions (Ag-0, Bor-1, Br-0, Ei-2, Got-7, Gu-0, Hr-5, Kin-0, Kondara, Ms-0, Mz-0, NFA-8, Nok-3, PNA-17, Rmx-A02, Sorbo, Sq-8, Uod-1, Wa-1, Yo-0) were obtained from the Supporting Information of Nordborg *et al.* (2005). Publicly available WGS for the same 20 accessions from Schmitz *et al.* (2013) were processed through the initial informed pipeline, and VQSR was performed using the stringently filtered variants from Cao *et al.* (2011) as a training set (prior of 7.0) and as a truth set. The resulting 95% tranche was used for BQSR as the WGS data for the 20 accessions were then processed through the informed pipeline. The Cao *et al.* (2011) variants were again used for VQSR. All alignments and variant calling were done against the version 10 *Arabidopsis* reference genome (*Arabidopsis* Genome Initiative *et al.* 2000).

To estimate error rates of the RIG workflow for WGS data, the resulting variant calls for the 20 accessions were compared to Sanger data from Nordborg *et al.* (2005) and variants from the Gramene database build 43, accessed January 2015 (Monaco *et al.* 2014). This requires the assumption that the Sanger data were 100% specific (*i.e.*, no false positives), and that the combination of the Sanger data and the Gramene build 43 variants were 100% sensitive (*i.e.*, no false negatives). Although the WGS data strongly suggest that these assumptions are false, this still provides a useful baseline for comparison; however, we expect that the true sensitivity and specificity achieved in this comparison are greater than the values obtained since false positives in the Sanger data translate to decreased sensitivity and false negatives in the Sanger data and Gramene build 43 translate to decreased positive predictive value. Genomic intervals used to evaluate performance were defined as a subset of the 861 intervals from Nordborg *et al.* (2005). Because many of the Sanger reads had an abundance of Ns at the beginning and end of the read, 50 bp from the ends of each interval were removed. Excluding intervals that did not have >90% of the bases covered at greater than 15 depth in all 20 WGS samples and >90% coverage in all 20 Sanger samples yielded 419 intervals that covered 200,887 bp of the genome.

Two of the accessions (Got-0 and Ms-0) were dropped from the comparison due to extensive disagreement between the Sanger variants and the WGS variants, potentially due to not truly being the same accession. We also found a number of sites that were heterozygous in the WGS accessions that that had been manually curated by Nordborg *et al.* (2005) to Ns in the Sanger data. Because this generates what appears to be a false positive in the WGS data, we used the Sanger data to identify false negatives, and variants from both the Sanger data and *Arabidopsis* variants contained in Gramene build 43 to identify false positives. Variants from the Nordborg *et al.* (2005) Sanger data contained in the designated genomic intervals but not contained in a tranche of the WGS data were considered false negatives for the purpose of calculating sensitivity. Variants contained in the WGS data but not in either the Nordborg *et al.* (2005) or the variants present in Gramene build 43 were considered false positives for the purpose of calculating positive predictive value. Variant site counts used for calculating sensitivity and positive predictive value are available in Table S4.

For the comparison, we report positive predictive value instead of specificity as a metric for false positives since the number of true negatives is far larger than the number of false positives, always leading to specificity values greater than 99.9%. As such, the performance of a tranche with a sensitivity of 95% and a positive predictive value of 99% is interpreted as a tranche where 95% of the true variants that existed were called and that 99% of the variants called are true variants.

### Code and hardware

Our implementation of the workflow and pipelines are available on GitHub at https://github.com/MulletLab/RIG as a series of Bash scripts to serve as an example, to provide the annotations we used for hard filtering and VQSR, and to list all of the additional software version numbers used. GATK’s Scala-based job submission controller, Queue, is suggested for implementing pipelines for the GATK for distributed computing resources; our implementation is in Bash because we experienced slowdowns in job submissions over time when using Queue (v3.1-1) on the Whole System Genomics Initiative cluster present at Texas A&M University.

## Results

### RIG: Recalibration and Interrelation of genomic sequence data with the GATK

The RIG workflow is a generalization of procedures to leverage existing genomic data when using the GATK v3.0+. Specifically, the workflow determines whether VQSR and/or BQSR are appropriate to perform, and the workflow iteratively constructs reliable variant resources for future use with the GATK. The procedures of the RIG workflow are divided into two phases: Phase I, where the entities necessary for workflow execution are defined (Figure 1), and Phase II, where those entities are used to execute the workflow (Figure 2).

#### RIG Phase I: define RIG entities:

Phase I of the RIG workflow defines the five entities necessary for execution of Phase II (Figure 1). The first entity is an ***analysis target***. The analysis target contains the sequence reads from which the user intends to call variants. Reads of the analysis target should be preprocessed by read-mapping, duplicate marking (if applicable), and indel realignment; this entity is depicted as a stack of BAM-format files in Figure 1. The second entity is a ***database of likelihoods***. The database of likelihoods contains the likelihood that a variant exists at a genomic position for all evaluated positions; this database consists of one or more gVCF-format flat files obtained from past GATK analyses of analysis targets produced by similar template preparation methods (*i.e.*, a database of likelihoods for WGS samples should not be used with an analysis target of reduced representation samples). This entity is depicted as a stack of circles in Figure 1, and it can be defined as empty. The third entity is a set of ***variant resources***. These are one or more files of VCF-format variant calls, and these calls should be conceptually (and physically, if necessary) partitioned into one or both of two categories: specific variant resources with low false positive rates, and sensitive variant resources with low false negative rates; a specific resource is necessary for VQSR and a sensitive resource is necessary for BQSR. As with the database of likelihoods, the variant resources can be empty and likely will be when first executing the workflow. The fourth and fifth entities can either be (i) constructed hypothetically based on a user’s expectations of the first three entities, or (ii) they can be empirically determined by performing the necessary analyses with the first three entities using the GATK. The fourth entity is a ***hypothetical database of likelihoods*** that is generated after adding the genotype likelihoods called from the analysis target to the existing database of likelihoods. The fifth entity is a set of ***shared variants***. Shared variants are variants contained in both the hypothetical database of likelihoods and in the chosen variant resources; shared variants can be specific, sensitive, or both (or empty) depending on the classification of the variant resource they were found in. Once all five entities are defined, the analysis target, the database of likelihoods, the variant resources, the hypothetical database of likelihoods, and the shared variants, a user can proceed to Phase II.

#### RIG Phase II: execute analysis:

The initial question of Phase II of the RIG workflow determines whether VQSR is appropriate based on the number of variants contained within the specific shared variants (RIG recommends at least 10,000 SNPs and 10,000 indels; Figure 2). A specific variant resource is required since false positives negatively impact the training of the Gaussian mixture models during VQSR, whereas false negatives have lesser effect. If the specific shared variants do not satisfy these criteria, then the RIG workflow enters the naive pipeline in which called variants are hard filtered using user-designated filtration criteria such as depth (Figure 3). Variants passing user-designated filtration criteria can then be added to the collection of variant resources. Once the naive pipeline has been used to analyze enough analysis targets, the collection of variant resources may be sufficiently large to answer yes to the initial question.

If the specific shared variants contain at least 10,000 SNPs and 10,000 indels, the next question addresses if the samples and variants in the database of likelihoods (if it is not empty) had previously undergone BQSR. If not, and if the reads corresponding to the samples used to generate the database of likelihoods are available, then the analysis target is updated with those reads, the database of likelihoods is set to empty, and the user returns to RIG Phase I (Figure 1) with the new analysis target and the empty database of likelihoods.

If the reads used in the construction of the database of likelihoods had previously undergone BQSR, or if the database of likelihoods is empty, then the final assessment determines whether the analysis target and the sensitive shared variants are appropriate for BQSR. A sensitive variant resource is necessary since false negatives cause BQSR to treat true variants in the analysis target as errors and will skew quality scores down, whereas false positives have a lesser chance to skew quality scores up. If BQSR is appropriate, the user follows the informed pipeline which emulates the GATK’s Best Practices (Van Der Auwera *et al.* 2013). If BQSR is not appropriate, the user first uses the initial informed pipeline in which VQSR is performed on the raw variants to generate a *temporary sensitive variant resource* which is used during the execution of the informed pipeline that immediately follows the initial informed pipeline (Figure 3).

Construction of variant resources and adding them to the collection of variant resources is the end step of any path through the RIG workflow (Figure 4). Deciding the criteria for generating the variant resources at the end is a highly user-driven process that should consider the specific properties of the analysis target. For example, we generate highly specific variant resources from experimental crosses based on markers that segregate as expected (see the section *Materials and Methods*). Additionally, the variant annotations used for hard filtering and VQSR should differ based on how reads should behave in the analysis target; that is, reduced representation data should not use the same annotations as WGS data for hard filtering and VQSR because reads are not distributed around variants in a similar manner. To provide an example, we discuss the methods we use to select VQSR tranches and construct variant resources in Figure 4 and in the section *Materials and Methods*. We also have made our code available on GitHub at https://github.com/MulletLab/RIG as an example and to provide the parameters and variant annotations we use.

### Interrelation of genomic data enables a specificity and sensitivity framework for variant calls

**Figure 5:**
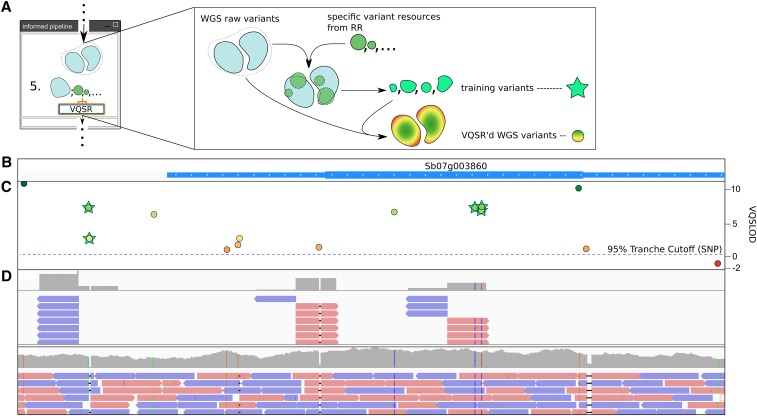
Interrelation of different genomic sequence data sources using the RIG workflow. (A) Schematic of how variants from reduced representation sequence (RR) data present in whole-genome sequence (WGS) data can be used to VQSR the WGS raw variants and assign VQSLOD scores to those variants. (B−D) Visualization of the genomic region of Sb07g003860, a gene involved in sorghum midrib coloration (Bout and Vermerris 2003). (B) the Sbi1.4 gene annotation; (C) shows the assigned VQSLOD scores for variants called in the region from WGS data; (D) shows the depth of coverage and mapped sequence reads for reduced representation and WGS data, respectively, for one sorghum line (BTx642). The RIG workflow enables variants called in the reduced representation sequence data to be used to inform and recalibrate the WGS analyses, and *vice versa*. This puts all of the variant calls into the GATK’s probabilistic framework whereby variants can be filtered based on their reliability. Users interested in more sensitive or specific call sets can choose more inclusive or exclusive tranches, respectively, by changing the cutoff indicated by the blue dotted line in Panel C. The common and standardized file formats emitted by the GATK enable downstream interoperability between analysis and visualization tools, such as the Integrative Genomics Viewer that produced (B) and (D) (Thorvaldsdóttir *et al.* 2012). RIG, Recalibration and Interrelation of genomic sequence data with the GATK; VQSR, Variant Quality Score Recalibration; VQSLOD, logarithm of odds ratio that a variant is real *vs.* not under the trained Gaussian mixture model; GATK, Genome Analysis Toolkit.

In accordance with the RIG workflow, we used reduced representation data of an experimental cross and association panels to enable both BQSR and VQSR of WGS data of 49 resequenced individuals for the crop plant *Sorghum bicolor*. By interrelating data sources produced by different template generation methods with the RIG workflow, we enforced that the variants used to train the VQSR Gaussian mixture models that determine a variant’s VQSLOD score (logarithm of odds ratio that a variant is real *vs.* not real under the trained Gaussian mixture model) were found orthogonally, providing additional confidence that the variants used for training were real variants. Additionally, the differences in reliability of the training variants due to the different experimental designs were also considered for training of the VQSR models; variants from the experimental cross were assigned a higher prior likelihood of being correct than those from the association panels. By following the RIG workflow, each SNP and indel in the raw WGS variants was assigned a VQSLOD score that reflects its reliability. Figure 5 depicts the process of interrelating data for VQSR and the resulting VQSLOD scores of variant calls. While interrelating data from different template generation methods may be optimal, we also obtained good performance by following similar processing logic using only *Arabidopsis* WGS data. In this way, the RIG workflow enables one of the greatest strengths of the GATK: the ability to put variant calls in a probabilistic framework that allows users to define where on the sensitivity and specificity spectrum the variants should sit for their target downstream application.

### Evaluation of recalibrated variants from the RIG workflow

Although a formal evaluation of the accuracy of variant calling pipelines remains unfeasible for nonsimulated sequence data (Li 2014), we estimated the performance of the workflow using both sorghum and *Arabidopsis* sequence data. For the sorghum data, we compared the variants called from sorghum WGS data via the RIG workflow to (i) a collection of reliable variants that were not used to train the VQSR models and (ii) a previously published sorghum variant calling analysis. We then used the sorghum WGS variants to recalibrate reduced representation data, and used the recalibrated variants for a genome-wide association study. Lastly, we further validated the performance of the RIG workflow using publicly available Sanger sequence and WGS data from *Arabidopsis*.

#### Evaluation of recalibrated sorghum variants:

First, we examined the overlap between the recalibrated sorghum WGS variants and a collection of reliable variants that were not used to train the VQSR models. This collection of reliable variants, hereafter referred to as the Independent-Family (IF) set, originated from a biparental cross genotyped using a reduced representation method; the IF set was obtained in a similar manner to the Family Reference Variant Resource that was used for training during VQSR, and the IF set represented a set of highly specific, genetically mappable variants (see the section *Materials and Methods*). Of the 10,737 SNPs and 3740 indels in the IF set, 10,557 SNPs and 3632 indels had also been called from the 49 WGS samples (of which 2 samples represented the parents of the biparental cross). The IF variants present in the recalibrated WGS variants had median VQSLOD scores of 8.22 and 5.29 for SNPs and indels, respectively, suggesting that the trained Gaussian mixture models correctly assigned true variants with highly positive VQSLOD scores (Figure S1, Table S1, and Table S2). Furthermore, the proportion of IF set variants that were also contained in the 95% and 75% tranches correspond to their respective tranche cutoffs, indicating that the tranche cutoffs were functioning as expected. Since tranche cutoffs represent the VQSLOD score over which a certain proportion of variants from the designated VQSR truth set will be retained, we expected the proportion of IF variants present in each tranche to approximate the tranche cutoff. As expected, proportions of the IF set retained in each tranche were similar to the tranche cutoff. For example, the 95% SNP tranche retained 97% of the SNPs in the IF set, and the 95% indel tranche retained 94% of the indels in the IF set (Table S2). These results indicate that the Gaussian mixture models for the WGS data were adequately trained and that the tranche cutoffs were functioning as expected.

**Figure 6:**
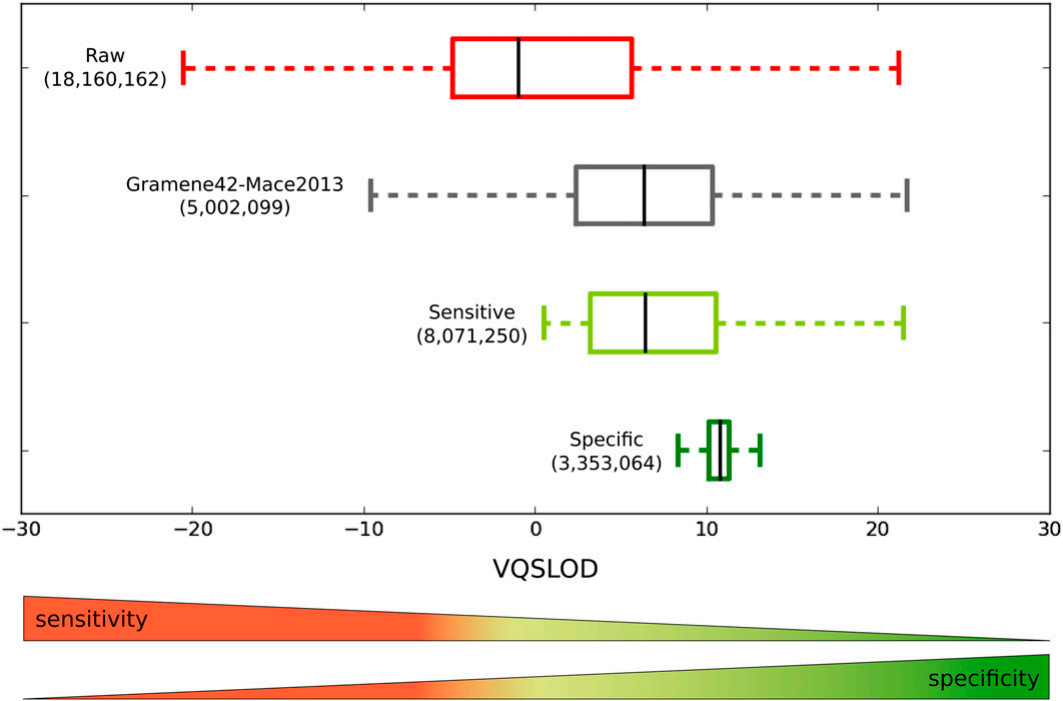
Comparison of VQSLOD score distributions for RIG-produced variant sets and a variant set from a previous study. VQSLOD (log of odds that a variant is real *vs.* not under the trained Gaussian mixture model) scores were calculated during VQSR of SNPs found in whole-genome sequence data using a Gaussian mixture model trained using SNPs originally found in reduced representation sequence data. For the 5,002,099 SNPs from Gramene42-Mace2013 that had been assigned VQSLOD scores in the Raw set produced by the RIG workflow, the median VQSLOD score is similar to the median of the 8,071,250 SNPs in the Sensitive set. The Sensitive set contains 1,620,622 more SNPs than the 6,450,628 SNPs in Gramene42-Mace2013, suggesting that the RIG-enabled VQSR allowed for a considerably more sensitive call set without a corresponding loss in specificity. VQSLOD, logarithm of odds ratio that a variant is real *vs.* not under the trained Gaussian mixture model; RIG, Recalibration and Interrelation of genomic sequence data with the GATK; VQSR, Variant Quality Score Recalibration; SNP, single-nucleotide polymorphism.

Second, we compared the recalibrated sorghum WGS variants to a previously published sorghum variant calling analysis. The previous study from Mace *et al.* (2013) called SNPs and indels from 47 sorghum WGS samples; the SNP calls were recently made available as part of Gramene build 42 (accessed September 2014), hereafter referred to as the Gramene42-Mace2013 set (Monaco *et al.* 2014). After excluding noncomparable variants from the calls produced by the RIG workflow (*i.e.*, indels, SNPs on super contigs, and variants not found in the 47 samples), we obtained a Raw set comprised of 18,160,612 SNPs. We constructed an additional two sets from this Raw set for comparison: the Sensitive set, derived from the 95% tranche and comprised of 8,071,250 SNPs, and the Specific set, derived from the 75% tranche and comprised of 3,353,064 SNPs. Of the 6,450,628 SNPs in Gramene42-Mace2013 set, 5,002,099 were present in the Raw set. It is difficult to conclusively attribute the 1,448,529 SNP difference to any specific factors, and high discordance between different variant callers is not uncommon (O’Rawe *et al.* 2013); we note that Mace *et al.* (2013) did not perform BQSR nor realignment around indels prior to calling SNPs, and they also used a different SNP calling algorithm. The overlapping 5,002,099 SNPs were used to compare the distribution of VQSLOD scores between the four sets (Figure 6). Because the VQSLOD score of all of the SNPs in the comparison were assigned under the same Gaussian mixture model and because the model was adequately trained as shown by the IF validation, comparisons of the relative sensitivity and specificity between the sets can be made. Given two sets of variants with similar VQSLOD distributions, the larger of the two sets contains more variants that are as likely to be true positives than the smaller set and is thus more sensitive. Furthermore, given two sets of variants where the VQSLOD distribution of one set contains a greater proportion of high VQSLOD score variants, the set with the greater proportion of high VQSLOD score variants contains variants that are more likely to be true positives and is thus more specific. As such, we find that the Raw set is the most sensitive but least specific; correspondingly, the Specific set is the most specific but least sensitive (Figure 6). The Sensitive set produced by the RIG workflow shows a dramatic improvement over the Gramene42-Mace2013 set in that it contains 1,620,622 more SNPs than the Gramene42-Mace2013 set while the median VQSLOD score remains similar with fewer negative VQSLOD scores, suggesting that the RIG workflow enabled greatly increased sensitivity without a corresponding loss in specificity.

As a final validation of the workflow with sorghum variants, we used a set of variants from reduced representation sequence data that had been recalibrated with WGS data to reproduce genome wide association results from the sorghum literature. There were 171 individuals contained within our reduced representation samples that had also previously been phenotyped as part of a sorghum association panel (Brown *et al.* 2008). After recalibrating the reduced representation data with the WGS data, we used the genotypes for these 171 individuals and phenotypes from Brown *et al.* (2008) to calculate genome wide associations (Figure S2 and Table S3) and reproduced known sorghum height QTL (Morris *et al.* 2013; Higgins *et al.* 2014). As such, the recalibrated reduced representation variants produced by the RIG workflow are useful for common downstream analyses, and these analyses are readily executable due to the GATK’s use of standard file formats.

#### Evaluation of recalibrated Arabidopsis variants:

Some organisms may not have sufficient data available from different template preparation methods to execute the RIG workflow as we did for sorghum. As such, we validated the performance of the RIG workflow using only WGS data as both the source of reliable variants and the analysis target. Efficacy of RIG was determined by comparing the variant calls produced by RIG from publicly available *Arabidopsis* WGS data against a collection of known variants from Sanger sequence data and variants present in the Gramene database (build 43; accessed January 2015) (Nordborg *et al.* 2005; Cao *et al.* 2011; Schmitz *et al.* 2013; Monaco *et al.* 2014).

The comparison used variant calls from 419 genomic intervals spanning 200,887 bp (containing at least 2,850 SNP and 375 indels) for 18 *Arabidopsis* accessions. Variants from the Sanger sequence data not present in the RIG variants were considered false negatives, and RIG variants not present in either the Sanger data or the Gramene build 43 set were considered false positives; these values were used to estimate sensitivity and positive predictive value of multiple tranches produced with RIG from *Arabidopsis* WGS data (Table 1 and Table S4). This yielded a conservative estimate of RIG variant calls whereby 95% sensitivity and 99% positive predictive value are achieved in one tranche with the RIG workflow (the 99.0% tranche in this case). As shown in the sorghum data, larger percentage tranches are more sensitive but less specific; smaller percentage tranches are less sensitive but more specific. The optimal choice of tranche will, again, depend on the downstream application for which the variant set will be used. We note that the sensitivity does not correspond with the tranche cutoffs as well as they did in the sorghum validation; this may be a result of the low sensitivity of the Sanger variants due to manual removal of variant calls by Nordborg *et al.* (2005) during data curation. Ultimately, this *Arabidopsis* validation in combination with the sorghum validation demonstrates that the RIG workflow can produce accurate call sets from a variety of genomic data sources.

**Table 1:** Performance of tranches from *Arabidopsis* WGS sequence data

Tranche, %	Sensitivity, %	Positive Predictive Value, %
100.0	99.9	93.7
99.9	99.3	95.4
99.0	94.9	99.2
97.5	92.0	99.3
95.0	89.3	99.4
75.0	54.3	99.6

Sensitivity and positive predictive value of multiple tranches of recalibrated variants from *Arabidopsis* WGS data were calculated using variants found in Sanger sequence data from Nordborg *et al.* (2005) for sensitivity; variants found in both the Sanger sequence data and in Gramene (build 43) were used to estimate positive predictive value (Table S4). For simplicity, the tranche percentage corresponds to both the SNP and the indel tranche. We note that these values are not generally applicable to other RIG analyses and these should not be taken as representative of how tranches in other analyses will behave; tranches should be chosen based on the reliability of the variants designated as truth for VQSR. WGS, whole-genome sequencing; SNP, single-nucleotide polymorphism; RIG, Recalibration and Interrelation of genomic sequence data with the GATK; VQSLOD, logarithm of odds ratio that a variant is real *vs.* not under the trained Gaussian mixture model; VQSR, Variant Quality Score Recalibration.

## Discussion

The GATK has been shown to outperform other variant calling methods in benchmarking studies, and the RIG workflow enables the analysis benefits afforded by the GATK to research communities lacking validated variant resources (Liu *et al.* 2013; Pirooznia *et al.* 2014). RIG also provides access to features absent in current reduced representation sequence data analysis platforms. Two popular reduced representation sequence data analysis solutions, TASSEL and Stacks, are highly specialized for their respective data sources (GBS and RAD-seq, respectively), and they perform well in their target domains; however, they lack features that readily allow the interrelation of WGS with reduced representation sequence data, as well as the ability to accurately call indels (Catchen *et al.* 2011; Glaubitz *et al.* 2014). RIG provides a means to access both of these features, as well as benefit from accuracy gains from BQSR, joint genotyping, and VQSR. For organisms with a reference genome, the RIG workflow stands as a useful analysis alternative applicable to both reduced representation and WGS data, and RIG is also readily applicable to exome and RNA-seq data due to the GATK’s flexibility. However, because the GATK, and by extension, RIG, cannot operate without a reference genome, software like TASSEL and Stacks will continue to fill important analysis roles, although this may change if software like dDocent, which allows users to take advantage of some of the GATK’s benefits even in the absence of a complete reference genome, gain adoption (Puritz *et al.* 2014). Ultimately, RIG was developed in the context of a genetics lab seeking accurate variant calls from multiple sequence data sources for agriculturally important organisms with a reference genome, and we expect it will be beneficial to those with similar use cases.

The RIG workflow requires that the shared variants are comprised of 10,000 SNPs and 10,000 indels for VQSR; however, the GATK developers have successfully used considerably fewer to good effect (Depristo *et al.* 2011). We chose 10,000 for both SNPs and indels as the requirement because we have obtained useful results using these values; the values are not a hard rule. As such, the user can construct their own values by evaluating the VQSR and BQSR reports produced by the GATK to determine whether (i) the Gaussian mixture models were adequately trained to distinguish between variants of differing reliability, and (ii) whether the empirically determined base quality score recalibrations appear reasonable for the sequencing platform.

In cases in which sequence data from different template preparation methods are not available, it will not be possible to identify shared variants from orthogonal approaches as we did with sorghum sequence data. We ensured the variants designated as truth for VQSR originated from an analysis target produced by a different method (*e.g.*, variants found in reduced representation data were used for VQSR of a WGS analysis target). This enforced that variants used in VQSR were found in two independent template preparation methods to approximate variants found using orthogonal methods. Since such genomic resources may not always be available, we also evaluated performance of a use case where only WGS data were available, and we showed that high levels of sensitivity and positive predictive value can be achieved using only WGS data. In cases in which the analysis target is the only source of variants, we and other GATK users have had some success by taking the analysis target through the naive pipeline, hard filtering to generate a temporary sensitive variant resource, and using that temporary sensitive variant resource to BQSR the analysis target. This procedure is iteratively repeated until the BQSR results from the current and preceding iteration converge, and then a specific variant resource is generated by stringently hard filtering to use as a bootstrapped variant resource in VQSR. This ultimately skews VQSR based on the annotations used to hard filter the variants during bootstrapping, but communities lacking sufficient data sources may find this procedure to be an acceptable alternative.

The RIG workflow enables research communities to use the GATK (i) to interrelate different sequencing template preparation methods such as reduced representation and WGS into common, standardized file formats; (ii) accurately call genetic variants from genomic sequence data; and (iii) to iteratively refine variant resources. The RIG workflow will contribute to progress in construction of more complete catalogs of genetic variation, and the ability to readily interrelate variants from different sequence data sources using the GATK will increase the rate at which variants associated with a phenotype lead to the identification of the genetic variation that causes the phenotype.

## 

## Supplementary Material

Supporting Information
