# Supplementary material for: RIG: Recalibration and Interrelation of Genomic Sequence Data with the GATK
Source: G3 (Bethesda). 2015 Feb 13;5(4):655–65. doi: 10.1534/g3.115.017012 (PMC4390580; doi:10.1534/g3.115.017012)
Supplement: Supporting Information [file supp_5_4_655__index.html]

RIG: Recalibration and Interrelation of Genomic Sequence Data with the GATK — RIG: Recalibration and Interrelation of Genomic Sequence Data with the GATK — Supporting Information 

# RIG: Recalibration and Interrelation of Genomic Sequence Data with the GATK

## Supporting Information for McCormick, Truong, and Mullet, 2015

**Files in this Data Supplement:**

- Supporting Information - Figures S1-S2, Tables S1-S4, and Literature Cited (PDF, 1 MB)
- Table S1 - Recovery of variants in the Independent-Family set within the WGS sets. (PDF, 53 KB)
- Table S2 - Comparison of the Independent-Family set with WGS tranches. (PDF, 53 KB)
- Figure S1 - Distributions of VQSLOD scores for variants from the WGS Raw set that were also contained in the Independent-Family (IF) set. (PDF, 155 KB)
- Figure S2 - Genome-wide associations for preflag leaf height using RIG-generated variants called from reduced representation data. (PDF, 1 MB)
- Table S3 - Comparison GWAS results from RIG-generated variants to previously reported results. (PDF, 71 KB)
- Table S4 - Variant site counts used to calculate sensitivity and positive predictive value for each tranche. (PDF, 88 KB)
