# Supplementary material for: RIG: Recalibration and Interrelation of Genomic Sequence Data with the GATK
Source: G3 (Bethesda). 2015 Feb 13;5(4):655–65. doi: 10.1534/g3.115.017012 (PMC4390580; doi:10.1534/g3.115.017012)
Supplement: Supporting Information [file supp_g3.115.017012_017012SI.pdf]

G3 · Genes | Genomes | Genetics

RIG: Recalibration and Interrelation of genomic sequence data with the GATK

Ryan F. McCormick*,[$], Sandra K. Truong*,[$], and John E. Mullet*,[$,†]
* Interdisciplinary Program in Genetics, Texas A&M University, College Station, Texas 77843
[$] Biochemistry & Biophysics Department, Texas A&M University, College Station, Texas 77843
[†] Corresponding author

John E. Mullet
300 Olsen Boulevard
College Station, TX 77843-2128
(979) 845 0722
jmullet@tamu.edu

DOI: 10.1534/g3.115.017012

Table S1: **Recovery of variants in the Independent-Family set within the WGS sets.** The table shows the intersection of variants between the Independent-Family (IF) set and the Raw, Sensitive, and Specific sets from 49 WGS samples. The IF set is comprised of genetically mappable variants from a biparental cross, and the Raw, Sensitive, and Specific sets correspond to the variant calls generated from 49 WGS samples at the 100%, 95%, and 75% tranches, respectively, for both the SNP and indel models. The Independent-Family set was not used to train the VQSR Gaussian mixture models that assigned VQSLOD scores to the WGS variants. Variants not recovered in the WGS Raw set can either be false positives in the IF set or false negatives in the Raw set. False negatives in the Raw set can occur if the variant did not have sufficient coverage in the WGS data. False positives in the IF set can occur if, in the reduced representation data, a true variant (e.g., an indel) caused errors in read mapping that produced an artifactual variant (e.g., a SNP); such an artifactual variant will segregate with the true variant and appear to be genetically mappable. While procedures like indel realignment should resolve these cases, the way reads stack and the high depth of some loci acheived with reduced representation methods can prevent accurate local reassembly. These data show that most of the variants from the reduced representation IF data are identified in the WGS data and that sensitivity decreases with descending tranches.

	**# SNPs**	**% SNP**	**# indels**	**% indel**
**Independent-Family (IF)**	10,737	100%	3,740	100%
**IF ∩ Raw**	10,557	98%	3,632	97%
**IF ∩ Sensitive**	10,211	95%	3,402	91%
**IF ∩ Specific**	7,966	74%	2,330	62%

Table S2: **Comparison of the Independent-Family set with WGS tranches.** The intersections of variants from the Independent-Family (IF) set with each of the WGS variant sets were compared (see Table S1). Assuming that the IF variants represent "true" variants, the tranche cutoffs are in good agreement with how many of the IF variants were present in the tranche (even though the IF variants were not used to train the VQSR Gaussian mixture models). For example, the 95% SNP tranche represents the minimum VQSLOD cutoff whereby 95% of the "true" variants provided to VQSR would be retained. Accordingly in our data, the 95% WGS SNP tranche contains 97% of the available IF set SNPs, suggesting that the models were appropriately trained and that the tranche cutoffs functioned as expected.

	**# SNPs**	**% SNPS**	**SNP tranche**	**min(VQSLOD of tranche)**
**IF ∩ Raw**	10,557	100%	100%	-39,962.6819
**IF ∩ Sensitive**	10,211	97%	95%	0.4462
**IF ∩ Specific**	7,966	75%	75%	7.1643
	**# indels**	**% indels**	**indel tranche**	**min(VQSLOD of tranche)**
**IF ∩ Raw**	3,632	100%	100%	-39,645.5822
**IF ∩ Sensitive**	3,402	94%	95%	1.1027
**IF ∩ Specific**	2,330	64%	75%	4.6878

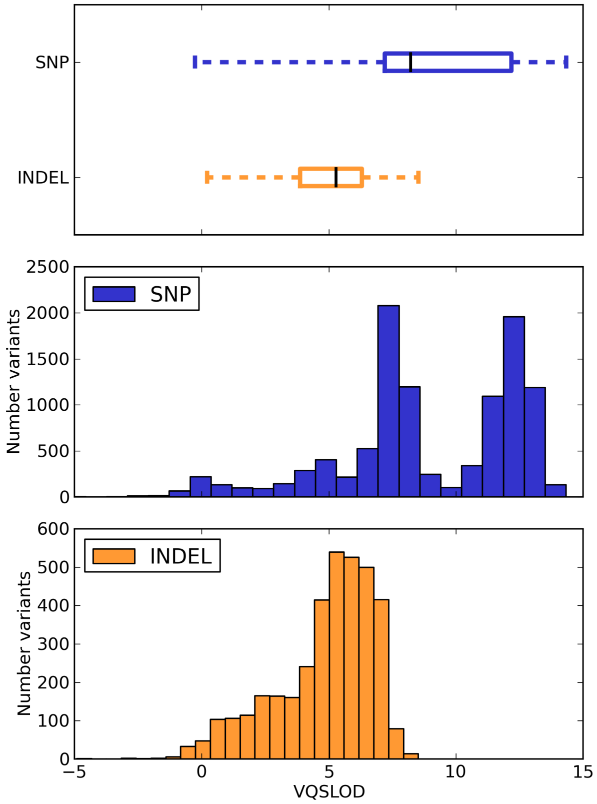

Figure S1: **Distributions of VQSLOD scores for variants from the WGS Raw set that were also contained in the Independent-Family (IF) set.** The VQSLOD distributions of the 10,557 SNPs and 3,632 indels from the WGS raw set that were also in the IF set are plotted here as box plots and as histograms (see Supplemental Tables S1 and S2). The median VQSLOD score of the SNPs and indels were 8.22 and 5.29, respectively, suggesting that the trained Gaussian mixture models correctly assigned true variants with positive VQSLOD scores. Variants from the IF set with low VQSLOD scores (e.g. < 0) potentially represent the false positives described in the caption of Supplemental Table S1 that were also called in the WGS data. Alternatively, they are true variants that did not receive sufficient coverage in the WGS data to provide strong evidence for their existence. The two peaks of the bimodal distribution of SNP VQSLOD scores correspond to whether or not certain variant annotations had been calculated by the GATK's HaplotypeCaller. Certain variant annotations, such as MQRankSum and ReadPosRankSum, are only calculated when a sample contains a mixture of reads displaying both the reference allele and the alternate allele for the variant; these annotations were typically not assigned to variants for which every sample was genotyped as homozygous. Both MQRankSum and ReadPosRank sum were used as annotations for training during VQSR; the lower VQSLOD peak consists mostly of variants assigned these annotations, and the larger VQSLOD peak consists mostly of variants that were not assigned these annotations. This suggests that these two annotations were often associated with less reliable variants in the resequenced sorghum lines which is expected given the inbred nature of most of the lines. A similar effect was seen with indels, though not as extreme.

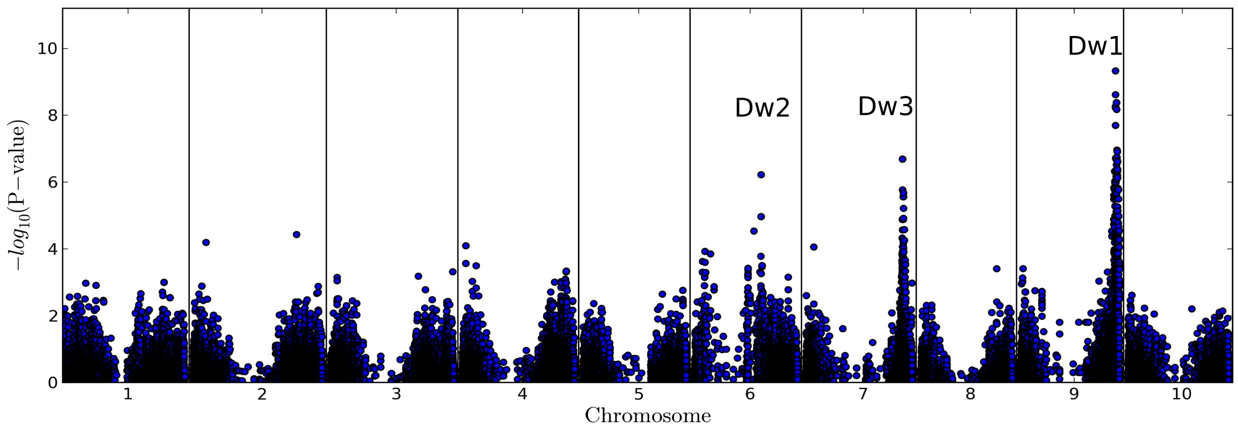

Figure S2: **Genome-wide associations for preflag leaf height using RIG-generated variants called from reduced representation data.** Of the 733 sorghum germplasm samples used to generate the Population Reference Variant Resource as part of the RIG workflow, 171 of the lines had been previously phenotyped by Brown *et al.* (2008). After producing a recalibrated, sensitive variant resource with the RIG workflow, missing genotypes were filled in using Beagle v4 release 1274 (Browning and Browning 2007). Variants were pre-processed (minor allele frequency $> 5\%$) and converted to PLINK binary format using PLINK v1.90-1 (Purcell and Chang 2014). The 171 phenotypes from Brown *et al.* (2008) were normalized using an Empirical Normal Quantile Transformation (ENQT) (Peng *et al.* 2007). Using GCTA v1.24.3, a genomic relationship matrix was generated and associations were calculated using GCTA's mixed linear model implementation (Yang *et al.* 2011). As shown in Supplemental Table S3, this analysis reproduced known QTL at the sorghum dwarfing loci Dw1, Dw2, and Dw3 on chromosomes 9, 6, and 7, respectively (Morris *et al.* 2013; Higgins *et al.* 2014).

Table S3: **Comparison GWAS results from RIG-generated variants to previously reported results.** The RIG column lists the position of the most significant marker identified by the GWAS described in Supplemental Figure S2. The Literature column lists the position of significant peaks reported by Morris *et al.* (2013) for Dw1 and Dw2 and the position of the cloned gene for Dw3 (Multani *et al.* 2003). Recalibrated variants identified from reduced representation sequence data using the RIG workflow are capable of reproducing known sorghum genome wide associations.

**Locus**	**Chromosome**	**RIG (Mbp)**	**Literature (Mbp)**
Dw2	6	40.2	39.7 - 42.6
Dw3	7	58.4	58.6
Dw1	9	57.2	57.2

Table S4: **Variant site counts used to calculate sensitivity and positive predictive value for each tranche.** Subsets of each of the six tranches (75.0%, 95.0%, 97.5%, 99.0%, 99.9%, and 100.0%) were used for determining sensitivity and positive predictive value. Sensitivity was calculated using $\frac{(Tranche \cap Nordborg)}{Nordborg}$. Positive predictive value was calculated using $\frac{(Tranche \cap Nordborg)+((Tranche \setminus Nordborg) \cap Gramene43)}{Tranche}$. For example, the sensitivity of the 75.0% tranche is $\frac{1762}{3243} = 0.543$ and the positive predictive value is $\frac{1762+20}{1789} = 0.996$

**Variant Source**	**Number Variant Sites**
Nordborg 2005	3243
75.0%	1789
75.0% ∩ Nordborg 2005	1762
(75.0% \ Nordborg 2005) ∩ Gramene43	20
75.0% \ (Nordborg 2005 ∪ Gramene43)	7
95.0%	3014
95.0% ∩ Nordborg 2005	2897
(95.0% \ Nordborg 2005) ∩ Gramene43	98
95.0% \ (Nordborg 2005 ∪ Gramene43)	19
97.5%	3107
97.5% ∩ Nordborg 2005	2982
(97.5% \ Nordborg 2005) ∩ Gramene43	103
97.5% \ (Nordborg 2005 ∪ Gramene43)	22
99.0%	3212
99.0% ∩ Nordborg 2005	3078
(99.0% \ Nordborg 2005) ∩ Gramene43	109
99.0% \ (Nordborg 2005 ∪ Gramene43)	25
99.9%	3589
99.9% ∩ Nordborg 2005	3220
(99.9% \ Nordborg 2005) ∩ Gramene43	205
99.9% \ (Nordborg 2005 ∪ Gramene43)	164
100.0%	3716
100.0% ∩ Nordborg 2005	3241
(100.0% \ Nordborg 2005) ∩ Gramene43	240
100.0% \ (Nordborg 2005 ∪ Gramene43)	235

LITERATURE CITED

Brown, P. J., W. L. Rooney, C. Franks, and S. Kresovich, 2008 Efficient mapping of plant height quantitative trait loci in a sorghum association population with introgressed dwarfing genes. Genetics **180**: 629–637.

Browning, S. R., and B. L. Browning, 2007 Rapid and accurate haplotype phasing and missing-data inference for whole-genome association studies by use of localized haplotype clustering. The American Journal of Human Genetics **81**: 1084–1097.

Higgins, R. H., C. S. Thurber, I. Assaranurak, and P. J. Brown, 2014 Multi-parental mapping of plant height and flowering time QTL in partially isogenic sorghum families. G3: Genes—Genomes—Genetics **4**: 1593–1602.

Morris, G. P., P. Ramu, S. P. Deshpande, C. T. Hash, T. Shah, *et al.*, 2013 Population genomic and genome-wide association studies of agroclimatic traits in sorghum. Proceedings of the National Academy of Sciences **110**: 453–458.

Multani, D. S., S. P. Briggs, M. A. Chamberlin, J. J. Blakeslee, A. S. Murphy, *et al.*, 2003 Loss of an MDR transporter in compact stalks of maize *br2* and sorghum *dw3* mutants. Science **302**: 81–84.

Peng, B., K. Y. Robert, K. L. DeHoff, and C. I. Amos, 2007 Normalizing a large number of quantitative traits using empirical normal quantile transformation **1**: S156.

Purcell, S., and C. Chang, 2014 PLINK. https://www.cog-genomics.org/plink2 .

Yang, J., S. H. Lee, M. E. Goddard, and P. M. Visscher, 2011 GCTA: a tool for genome-wide complex trait analysis. The American Journal of Human Genetics **88**: 76–82.
