# Supplementary material for: RIG: Recalibration and Interrelation of Genomic Sequence Data with the GATK
Source: G3 (Bethesda). 2015 Feb 13;5(4):655–65. doi: 10.1534/g3.115.017012 (PMC4390580; doi:10.1534/g3.115.017012)
Supplement: Supporting Information [file supp_g3.115.017012_TableS1.pdf]

Table S1: **Recovery of variants in the Independent-Family set within the WGS sets.** The table shows the intersection of variants between the Independent-Family (IF) set and the Raw, Sensitive, and Specific sets from 49 WGS samples. The IF set is comprised of genetically mappable variants from a biparental cross, and the Raw, Sensitive, and Specific sets correspond to the variant calls generated from 49 WGS samples at the 100%, 95%, and 75% tranches, respectively, for both the SNP and indel models. The Independent-Family set was not used to train the VQSR Gaussian mixture models that assigned VQSLOD scores to the WGS variants. Variants not recovered in the WGS Raw set can either be false positives in the IF set or false negatives in the Raw set. False negatives in the Raw set can occur if the variant did not have sufficient coverage in the WGS data. False positives in the IF set can occur if, in the reduced representation data, a true variant (e.g., an indel) caused errors in read mapping that produced an artifactual variant (e.g., a SNP); such an artifactual variant will segregate with the true variant and appear to be genetically mappable. While procedures like indel realignment should resolve these cases, the way reads stack and the high depth of some loci acheived with reduced representation methods can prevent accurate local reassembly. These data show that most of the variants from the reduced representation IF data are identified in the WGS data and that sensitivity decreases with descending tranches.

	**# SNPs**	**% SNP**	**# indels**	**% indel**
**Independent-Family (IF)**	10,737	100%	3,740	100%
**IF ∩ Raw**	10,557	98%	3,632	97%
**IF ∩ Sensitive**	10,211	95%	3,402	91%
**IF ∩ Specific**	7,966	74%	2,330	62%
