# Supplementary material for: RIG: Recalibration and Interrelation of Genomic Sequence Data with the GATK
Source: G3 (Bethesda). 2015 Feb 13;5(4):655–65. doi: 10.1534/g3.115.017012 (PMC4390580; doi:10.1534/g3.115.017012)
Supplement: Supporting Information [file supp_g3.115.017012_TableS2.pdf]

Table S2: **Comparison of the Independent-Family set with WGS tranches.** The intersections of variants from the Independent-Family (IF) set with each of the WGS variant sets were compared (see Table S1). Assuming that the IF variants represent "true" variants, the tranche cutoffs are in good agreement with how many of the IF variants were present in the tranche (even though the IF variants were not used to train the VQSR Gaussian mixture models). For example, the 95% SNP tranche represents the minimum VQSLOD cutoff whereby 95% of the "true" variants provided to VQSR would be retained. Accordingly in our data, the 95% WGS SNP tranche contains 97% of the available IF set SNPs, suggesting that the models were appropriately trained and that the tranche cutoffs functioned as expected.

	**# SNPs**	**% SNPS**	**SNP tranche**	**min(VQSLOD of tranche)**
**IF ∩ Raw**	10,557	100%	100%	-39,962.6819
**IF ∩ Sensitive**	10,211	97%	95%	0.4462
**IF ∩ Specific**	7,966	75%	75%	7.1643
	**# indels**	**% indels**	**indel tranche**	**min(VQSLOD of tranche)**
**IF ∩ Raw**	3,632	100%	100%	-39,645.5822
**IF ∩ Sensitive**	3,402	94%	95%	1.1027
**IF ∩ Specific**	2,330	64%	75%	4.6878
