# Supplementary material for: RIG: Recalibration and Interrelation of Genomic Sequence Data with the GATK
Source: G3 (Bethesda). 2015 Feb 13;5(4):655–65. doi: 10.1534/g3.115.017012 (PMC4390580; doi:10.1534/g3.115.017012)
Supplement: Supporting Information [file supp_g3.115.017012_FigureS1.pdf]

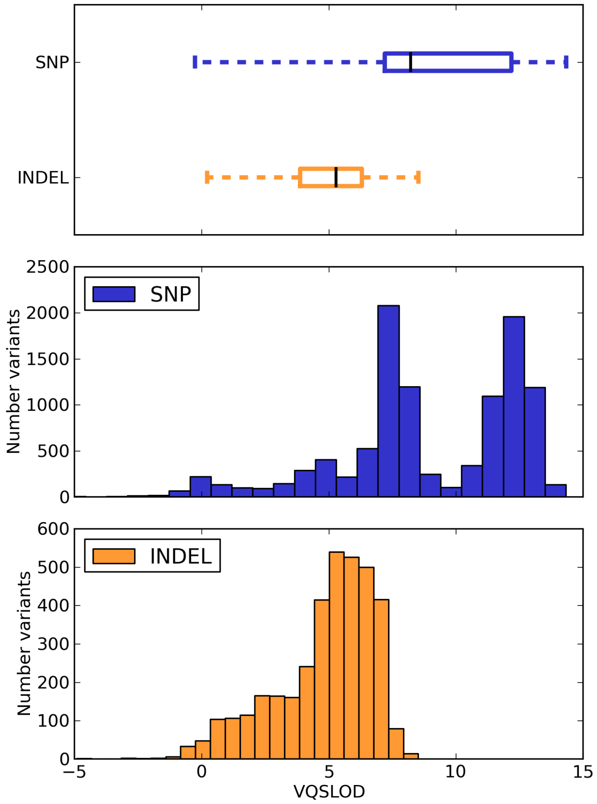

Figure S1: **Distributions of VQSLOD scores for variants from the WGS Raw set that were also contained in the Independent-Family (IF) set.** The VQSLOD distributions of the 10,557 SNPs and 3,632 indels from the WGS raw set that were also in the IF set are plotted here as box plots and as histograms (see Supplemental Tables S1 and S2). The median VQSLOD score of the SNPs and indels were 8.22 and 5.29, respectively, suggesting that the trained Gaussian mixture models correctly assigned true variants with positive VQSLOD scores. Variants from the IF set with low VQSLOD scores (e.g. < 0) potentially represent the false positives described in the caption of Supplemental Table S1 that were also called in the WGS data. Alternatively, they are true variants that did not receive sufficient coverage in the WGS data to provide strong evidence for their existence. The two peaks of the bimodal distribution of SNP VQSLOD scores correspond to whether or not certain variant annotations had been calculated by the GATK's HaplotypeCaller. Certain variant annotations, such as MQRankSum and ReadPosRankSum, are only calculated when a sample contains a mixture of reads displaying both the reference allele and the alternate allele for the variant; these annotations were typically not assigned to variants for which every sample was genotyped as homozygous. Both MQRankSum and ReadPosRank sum were used as annotations for training during VQSR; the lower VQSLOD peak consists mostly of variants assigned these annotations, and the larger VQSLOD peak consists mostly of variants that were not assigned these annotations. This suggests that these two annotations were often associated with less reliable variants in the resequenced sorghum lines which is expected given the inbred nature of most of the lines. A similar effect was seen with indels, though not as extreme.
