# Supplementary material for: RIG: Recalibration and Interrelation of Genomic Sequence Data with the GATK
Source: G3 (Bethesda). 2015 Feb 13;5(4):655–65. doi: 10.1534/g3.115.017012 (PMC4390580; doi:10.1534/g3.115.017012)
Supplement: Supporting Information [file supp_g3.115.017012_FigureS2.pdf]

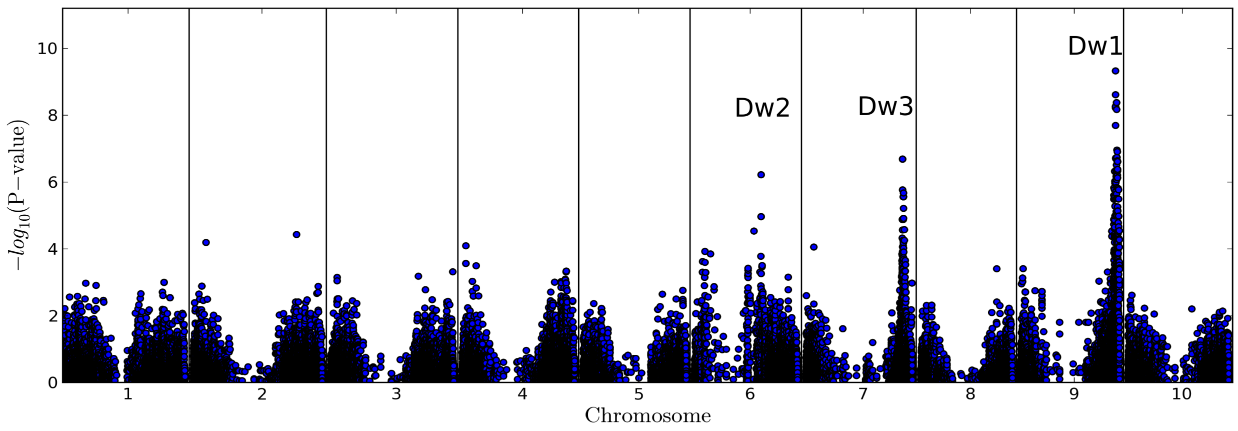

Figure S2: **Genome-wide associations for preflag leaf height using RIG-generated variants called from reduced representation data.** Of the 733 sorghum germplasm samples used to generate the Population Reference Variant Resource as part of the RIG workflow, 171 of the lines had been previously phenotyped by Brown *et al.* (2008). After producing a recalibrated, sensitive variant resource with the RIG workflow, missing genotypes were filled in using Beagle v4 release 1274 (Browning and Browning 2007). Variants were pre-processed (minor allele frequency > 5%) and converted to PLINK binary format using PLINK v1.90-1 (Purcell and Chang 2014). The 171 phenotypes from Brown *et al.* (2008) were normalized using an Empirical Normal Quantile Transformation (ENQT) (Peng *et al.* 2007). Using GCTA v1.24.3, a genomic relationship matrix was generated and associations were calculated using GCTA's mixed linear model implementation (Yang *et al.* 2011). As shown in Supplemental Table S3, this analysis reproduced known QTL at the sorghum dwarfing loci Dw1, Dw2, and Dw3 on chromosomes 9, 6, and 7, respectively (Morris *et al.* 2013; Higgins *et al.* 2014).
