# Supplementary material for: RIG: Recalibration and Interrelation of Genomic Sequence Data with the GATK
Source: G3 (Bethesda). 2015 Feb 13;5(4):655–65. doi: 10.1534/g3.115.017012 (PMC4390580; doi:10.1534/g3.115.017012)
Supplement: Supporting Information [file supp_g3.115.017012_TableS3.pdf]

Table S3: **Comparison GWAS results from RIG-generated variants to previously reported results.** The RIG column lists the position of the most significant marker identified by the GWAS described in Supplemental Figure S2. The Literature column lists the position of significant peaks reported by Morris *et al.* (2013) for Dw1 and Dw2 and the position of the cloned gene for Dw3 (Multani *et al.* 2003). Recalibrated variants identified from reduced representation sequence data using the RIG workflow are capable of reproducing known sorghum genome wide associations.

**Locus**	**Chromosome**	**RIG (Mbp)**	**Literature (Mbp)**
Dw2	6	40.2	39.7 - 42.6
Dw3	7	58.4	58.6
Dw1	9	57.2	57.2
