# Supplementary material for: RIG: Recalibration and Interrelation of Genomic Sequence Data with the GATK
Source: G3 (Bethesda). 2015 Feb 13;5(4):655–65. doi: 10.1534/g3.115.017012 (PMC4390580; doi:10.1534/g3.115.017012)
Supplement: Supporting Information [file supp_g3.115.017012_TableS4.pdf]

Table S4: **Variant site counts used to calculate sensitivity and positive predictive value for each tranche.** Subsets of each of the six tranches (75.0%, 95.0%, 97.5%, 99.0%, 99.9%, and 100.0%) were used for determining sensitivity and positive predictive value. Sensitivity was calculated using $\frac{(Tranche \cap Nordborg)}{Nordborg}$. Positive predictive value was calculated using $\frac{(Tranche \cap Nordborg)+((Tranche \setminus Nordborg) \cap Gramene43)}{Tranche}$. For example, the sensitivity of the 75.0% tranche is $\frac{1762}{3243} = 0.543$ and the positive predictive value is $\frac{1762+20}{1789} = 0.996$

Variant Source	Number Variant Sites
Nordborg 2005	3243
75.0%	1789
75.0% ∩ Nordborg 2005	1762
(75.0% \ Nordborg 2005) ∩ Gramene43	20
75.0% \ (Nordborg 2005 ∪ Gramene43)	7
95.0%	3014
95.0% ∩ Nordborg 2005	2897
(95.0% \ Nordborg 2005) ∩ Gramene43	98
95.0% \ (Nordborg 2005 ∪ Gramene43)	19
97.5%	3107
97.5% ∩ Nordborg 2005	2982
(97.5% \ Nordborg 2005) ∩ Gramene43	103
97.5% \ (Nordborg 2005 ∪ Gramene43)	22
99.0%	3212
99.0% ∩ Nordborg 2005	3078
(99.0% \ Nordborg 2005) ∩ Gramene43	109
99.0% \ (Nordborg 2005 ∪ Gramene43)	25
99.9%	3589
99.9% ∩ Nordborg 2005	3220
(99.9% \ Nordborg 2005) ∩ Gramene43	205
99.9% \ (Nordborg 2005 ∪ Gramene43)	164
100.0%	3716
100.0% ∩ Nordborg 2005	3241
(100.0% \ Nordborg 2005) ∩ Gramene43	240
100.0% \ (Nordborg 2005 ∪ Gramene43)	235
